# A crowdsourcing approach to understand weight and weight loss in men

**DOI:** 10.1016/j.pmedr.2018.12.004

**Published:** 2018-12-12

**Authors:** Tiffany Rounds, Josh Bongard, Paul Hines, Jean Harvey

**Affiliations:** a University of Vermont, Department of Nutrition and Food Sciences, Marsh Life Sciences 234, Burlington, VT 05405, United States; b University of Vermont, Department of Computer Science, Farrell Hall 205, Burlington, VT 05405, United States; c University of Vermont, Department of Electrical and Biomedical Engineering, Votey Building 315, Burlington, VT 05405, United States; d University of Vermont, Department of Nutrition and Food Sciences, Marsh Life Science 250, Burlington, VT 05405, United States

## Abstract

• Crowdsourcing can be used to detect unexpected barriers to male weight loss.
• Some unique behaviors related to high BMI were revealed including watching others play video games.
• Novel behaviors to target: less watching video games and more organized physical activity

## 1 Introduction

Obesity and overweight are key contributors to chronic disease and pose a large public health challenge, with approximately 74% of American men classified as overweight or obese (Flegal et al., 2012). In the United States in 2008, the medical costs associated with obesity reached $147 billion, and annual estimated medical costs are currently $1500 higher for individuals with obesity (Finkelstein et al., 2009). In 2013, approximately $2.4 billion was spent on weight loss services provided by over 29,000 companies, and 85% of consumers of the services were women (IBISWorld, 2013).

Men who are overweight or obese are generally recognized as a hard to involve, yet high-risk group for obesity-related chronic disease treatment (Morgan et al., 2011a; Young et al., 2012). A variety of studies have demonstrated that men are less likely to perceive themselves as overweight, and therefore are less likely to attempt weight loss or participate in a weight loss program (Andersson and Rössner, 1997; Brown et al., 2015; Collins et al., 2011; Lemon et al., 2009; Morgan et al., 2011a; Young et al., 2012). In a systematic review of weight loss interventions conducted online, notably <23% of the 5700 participants were men (Neve et al., 2010). However, while both qualitative and quantitative studies suggest limitations to the current literature related to weight loss interventions, there is less information on why men may be reluctant to seek out weight loss treatment in the first place. This illustrates a visible and pressing need to identify novel approaches and program elements that can effectively engage men in initial weight loss and successful long-term weight maintenance. Crowdsourcing has the ability to generate large amounts of data from a broader and more diverse population of men, thus ameliorating some of the limitations and bias inherent in other previously collected data.

Crowdsourcing is a strategic model used to draw insights from an interested group of individuals who are able to suggest solutions beyond those offered by traditional forms of research. In other words, the crowd “solves” the problem that has scientific professionals puzzled (Howe, 2006). Web-based crowdsourcing is an inexpensive, fast method to build new hypotheses and uncover unforeseen problems that experts may have previously overlooked (Swan, 2012). Crowdsourcing is a mixed methods approach with a form of qualitative methodology (the questions being submitted by the subjects) and a quantitative component (the numerical scoring of the answers to posed questions). Therefore, crowdsourcing provided the ideal methodology to use in this study where professionals are “stumped” about the issues surrounding male participation in weight loss interventions and are looking to generate new questions that haven't come with a more traditional type of qualitative research methodology. The goal of the study was to utilize crowdsourcing to detect possible unexpected or new predictors of barriers to weight loss in men in order to guide future intervention design and successful recruitment of men to a weight loss study.

## 2 Materials and methods

### 2.1 Recruitment

Participants were recruited for participation in this study through direct email from investigators, advertisements posted in a widely distributed University of Vermont email newsletter, and reddit.com, which is a social networking and news website with user-submitted content. The notice on reddit.com was posted in a specific section focused on weight loss www.reddit.com/r/loseit where many users spend time reading and commenting on other users' posts, links and photos. Reddit.com was specifically chosen due to the interactive nature of the website, the high number of users (approximately 900,000 unique visits each day), and the fact that 71% of Reddit users are men (Barthel et al., 2016).

### 2.2 Crowdsourcing methodology

The website used in this study was based on two prior experiments designed to study individual body mass index and the monthly electric energy consumption of a homeowner. While body mass index and energy consumption are not directly linked, the methodology employed and the two crowdsourcing websites operated in similar ways. These websites were reconstructed to collect crowdsourced predictors of BMI for men only (Bongard et al., 2013). The survey was designed so users could answer questions and pose new questions they believed could predict obesity. Our goal was to gather men's and women's insight about predictors, challenges, barriers and aspects related to male weight loss as well as investigate the relationship between male BMI and the answers to the crowdsourced survey questions. To provide a more interactive experience, users were asked to enter their real BMI at the beginning of the survey and the computer displayed their “predicted” BMI after each question was answered, based on the association of other users' responses and their self-reported BMI. Research participants who visited the website, titled “The Great Weight Debate”, were first asked to enter their height, weight and gender in order to calculate body mass index (BMI) and track gender for data collection purposes. While this study was specifically interested in predictors of male BMI, women were not prevented from answering and posing questions. However, female responses were excluded from all analyses. We permitted women to ask and answer questions in order to gather more potentially useful information (Do women have ideas about male weight loss that men perhaps have not considered?) We were specifically interested in the relationship between the survey questions and male BMI, which was why female responses were not included in the analyses. Users also had the opportunity to create a “profile” using their email address in order to be eligible for one of the three lottery-selected financial incentives ($100 VISA gift card) designed to encourage participants to answer all questions on the website at the time of their visit. Previous studies suggested the importance of an incentive to encourage users to answer more questions in the survey (Bevelander et al., 2014; Bongard et al., 2013).

### 2.3 Crowdsourcing survey

The home page for the survey provided a brief introduction to crowdsourcing and our research project, as well as a quick video demonstrating how to navigate the website. Contact information for the Principal Investigator and a ‘Frequently Asked Questions’ link were also available.

The survey was ‘seeded’ with five questions the investigators expected to be related to male BMI, based on previous research (Morgan et al., 2011b; Morgan et al., 2011c; Morgan et al., 2012). The seed questions were “Have you ever been diagnosed with diabetes?”, “Have you ever been diagnosed with high blood pressure?”, “Are you married?”, “Do you participate in an organized sports league?” (all yes/no responses) and, “How concerned are you with your appearance? (5 point Likert scale with 1 = not at all concerned and 5 = very concerned)”. All questions were given to participants in random order. Throughout the survey each question screen displayed the participant's actual BMI alongside their predicted BMI. The predicted BMI was calculated in real-time by performing linear regression on all of the questions and responses from earlier survey users and was updated each time the participant answered a question.

**Fig. 1:**
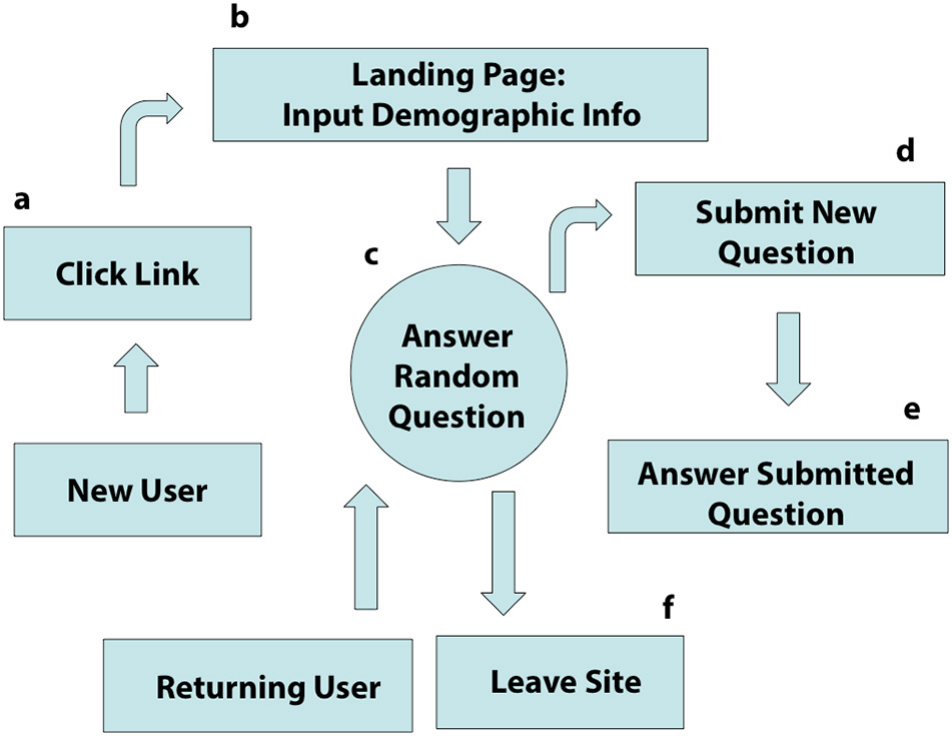
Crowdsourcing survey flow chart.

Users were able to pose questions of their own, that they believed would help predict male BMI, at any time throughout the survey with one of three different response formats: yes/no, a Likert scale rating 1–5, or a numerical answer. They were unable to pose open-ended questions, for data collection purposes. The survey monitor reviewed all suggested questions and approved questions were added to the survey expeditiously to be answered by other participants visiting the site. Questions were not approved for the survey if they were duplicates of questions already in the survey, contained profanity, or were not deemed to be serious (e.g., “Can you crush an entire bag of cheese doodles in one sitting?”) All questions were presented to users randomly, each with an equal chance of appearing for the user to answer. Fig. 1 outlines the crowdsourcing survey format as described by Bevelander and colleagues in a crowdsourcing study for childhood predictors of adult obesity (Bevelander et al., 2014).

Data were collected for a two-week cycle in December 2015. Similar to previous studies, no target sample size was established, as it is impossible to estimate the number of participants or the number of questions and answers collected (Bevelander et al., 2014; Bongard et al., 2013). For this study, the two-week fixed time frame was established during pre-study design and the sample size was the number of participants during that period.

### 2.4 Statistical analysis

Correlations between question responses and BMI were calculated between self-reported BMI and responses to questions for all male respondents. Pearson correlations were calculated for Likert scale and numerical responses and Spearman's rho was calculated for yes/no responses. All questions that received fewer than 50 responses were excluded from analyses due to insufficient response numbers to appropriately assess correlations.

Anonymity is one of the advantages of the crowdsourcing mechanism, however this introduces a level of reporting bias in that survey users may not accurately represent themselves in the study (providing incorrect BMI information, untruthfully answering questions, etc.) After collecting all questions and responses, we encountered six obviously falsified answers (responses with numbers that were statistical outliers, e.g. 1261 servings of dairy consumed on an average day), which were removed from all analyses.

## 3 Results

Five hundred twenty-two visitors initiated the survey during the two-week survey period. Males comprised 57% of respondents, therefore the sample size for data analysis was 298, once we removed participants who reported an implausible BMI. Men with obesity (BMI > 30) comprised 43.3% (n = 129) of the sample, overweight (BMI 25.0–29.9) 33.6%, (n = 100) healthy weight (BMI 18.5–24.9) 23.1%,(n = 69) and underweight (BMI < 18.5) 0%.

In addition to the five ‘seed’ questions supplied by the researchers, participants proposed 192 new questions, 188 of which were approved and added to the survey. In total, participants provided 21,846 responses to the 193 questions. Participants could only answer each question once. On average, each question was answered 126 times. Twenty-six questions were excluded due to fewer than 50 responses.

Out of the total 193 questions that were posted to the survey, 37 questions were significantly correlated with self-reported BMI (*p* < .05), 21 of which were significant to *p* < .01. Table 1 presents those questions scaled from the highest correlation to the lowest. The two most highly correlated predictors of high BMI were “Do you think your BMI is above average?” and “How many servings of dairy products do you typically consume on an average day?” The most highly correlated predictors of healthy BMI were “Are you happy with your weight?” and “Are you happy with your body?”

**Table 1:** Questions significantly correlated with BMI for men (highest correlations shown).

Question	Correlation	Number of responses	*p*-Value
Likert scale			
I enjoy watching home improvement shows.	0.221	95	0.032
I prefer salty snacks over sweet snacks.	0.174	153	0.032
I am comfortable in a swimsuit.	−0.302	164	<0.001
I buy organic even if it is more expensive.	−0.172	151	0.035
I always choose the healthy snack when given a choice.	−0.170	170	0.027
Yes/no			
Do you think your BMI is above average?	0.623	151	<0.001
Is anyone in your immediate family overweight?	0.341	154	<0.001
Have you lost weight and regained all or some of it?	0.313	98	0.002
Have you ever been diagnosed with high blood pressure?^a^	0.257	172	0.001
Have you tried home workout videos or programs?	0.242	100	0.015
Are you concerned with calorie and fat content of the foods you eat?	0.212	164	0.006
Do you own a cat?	0.199	130	0.023
Do you own a car?	0.188	115	0.045
Do you own a pet?	0.160	163	0.041
Are you happy with your weight?	−0.487	163	<0.001
Are you happy with your body?	−0.446	159	<0.001
Can you do a pullup?	−0.418	105	<0.001
Do you feel healthy?	−0.401	154	<0.001
Can you run 1 mile or further?	−0.316	106	0.001
Do you frequently listen to audiobooks or podcasts?	−0.284	84	0.009
Do you always take the stairs?	−0.268	109	0.005
Did you grow up in a family that embraced an active lifestyle?	−0.258	155	0.001
Do you plan active outdoor activities (such as going for a hike or going skiing) for fun?	−0.251	145	0.002
Can you do 10 pushups?	−0.234	117	0.014
Do you eat cereal for breakfast?	−0.215	153	0.008
Do you enjoy talking about food and fitness topics?	−0.203	113	0.031
Do you participate in an organized sports league?	−0.184	179	0.017
Do you participate in outdoor sports in the winter?	−0.180	143	0.032
Do you like to exercise when you are on vacation?	−0.164	160	0.038
Numerical			
How many servings of dairy products do you typically consume on an average day?	0.375	156	<0.001
How many diet sodas do you drink each day?	0.263	158	0.001
How many siblings do you have?	0.228	151	0.005
How many hours a week do you watch video games?	0.227	154	0.005
How many times a year do you eat at a buffet restaurant?	0.212	153	0.009
Approximately how many books do you read monthly?	0.167	153	0.039
How often a week do you eat a leafy green vegetable?	0.166	148	0.043
How many times a week do you eat ice cream/frozen yogurt/gelato?	−0.190	139	0.025

a Seed questions submitted by the study team.

Table 2 shows some of the most frequently answered questions, categorized by popular themes and sorted for significance.

**Table 2:** Crowdsourced questions answered grouped by category and statistical significance.

Category	Significant	Not significant
Eating habits	(+) How many servings of dairy products do you typically consume on an average day?	Do you drink milk?
	(−) Do you eat cereal for breakfast?	Do you always eat breakfast?
	(+) How many times a year do you eat at a buffet restaurant?	How many times a week do you eat out?
	(−) I always choose the healthy snack when given a choice.	Do you eat snacks while watching television?
	(+) How many diet sodas do you drink each day?	Do you follow a vegetarian diet?
Personal appearance/perception	(+) Do you think your BMI is above average?	Do you think how you look is important?
	(−) Are you happy with your body?	How concerned are you with your appearance?^a^
	(−) Are you happy with your weight?	I read magazines with pictures of men that look healthy.
	(−) I am comfortable in a swim suit.	How many times a day do you think about your weight?
	(−) Do you feel healthy?	
Built environment	(+) Do you own a car?	Do you live in an urban area?
		Do you live in a rural area?
Physical activity	(−) Do you participate in an organized sports league?^a^	Do you prefer to be with someone when exercising?
	(−) Do you plan active outdoor activities (such as going for a hike or going skiing) for fun?	I seek out exercise because it makes me both feel and look better.
	(−) Do you like to exercise when you are on vacation?	I don't seek out exercise because I don't have the time or energy.
	(−) Do you participate in outdoor sports in the winter?	You are more likely to exercise in a group setting.
	(−) Do you always take the stairs?	Do you use a standing desk?
Childhood/family	(+) How many siblings do you have?	My mother believes that exercise is important.
	(+) Is anyone in your immediate family overweight?	
	(−) Did you grow up in a family that embraced an active lifestyle?	
Medical issues	(+) Have you ever been diagnosed with high blood pressure?^a^	Have you ever suffered from depression or symptoms of depression?
		Is there any history of heart disease in your family?
		Do you see a doctor for an annual physical?
Non-eating/drinking routines	(−) Do you enjoy talking about food and fitness topics?	How many hours a night do you sleep?
	(+) I enjoy watching home improvement shows.	Do you regularly set goals for yourself?
	(+) Approximately how many books do you read monthly?	How many times per week do you weigh yourself?
	(+) How many hours a week do you watch video games?	How many hours of sports do you watch a week?
	(+) Do you own a cat?	Do you take vitamins or supplements on a daily basis?
	(−) Do you frequently listen to audiobooks or podcasts?	
Relationships/sex		Are you married?^a^
		Does your partner share the same diet goals as you?
		How many times a week do you have sex?
		How many hours of porn are you watching a week?

Note: (+) indicates positive correlation with BMI, (−) indicates negative correlation with BMI.

a Seed questions submitted by the study team.

## 4 Conclusions

Findings from this study demonstrated that the crowd was able to suggest many well-documented factors related to BMI. For example, prior obesity research suggests that many overweight or obese individuals are concerned about their appearance, body image and health (Drewnowski and Yee, 1987; Ginis et al., 2012; Schwartz and Brownell, 2004). This was also true of men in our study who were significantly more likely to answer (and ask) questions such as “Are you happy with your body” and “I am comfortable in a swimsuit.” Additionally, survey users suggested a variety of other factors known to be associated with weight including physical activity (e.g. participating in organized sports, planning outdoor activities, walking or biking to work), dietary intake (e.g. diet soda, breakfast, snacking habits), and screen time (e.g. television viewing, video games) (Banks et al., 2011; Fox and Hillsdon, 2007; Hu and Malik, 2010; Jeffery and French, 1998; Schlundt et al., 1992).

By contrast, several unexpected finding emerged. Several questions were related to dairy product consumption, but many did not support previous research. Number of reported daily dairy servings was positively significantly correlated with BMI, which could make sense if individuals are consuming high fat, high calorie dairy products. Some previous research found that high dairy product consumption was associated with lower body fatness (Davies et al., 2000; Zemel and Miller, 2004) although recent consensus is that dairy consumption alone has no substantive impact on weight one way or the other (Chen et al., 2012; Wang et al., 2014). Curiously, reported weekly consumption of ice cream/frozen yogurt/gelato was negatively significantly correlated with BMI and milk consumption was not significantly correlated at all. The questions asking participants about dietary intake were not validated ways to assess dietary intake; therefore, conclusions about the relationship between actual dietary intake and body weight cannot be made using this crowdsourcing approach. Regardless, dairy product consumption, rather than say, meat intake, was asked and answered by men.

Some unique, potentially sedentary activities (watching home improvement shows, reading books, watching video games) posed by men were correlated with higher BMI. While moderating the comment stream on reddit.com, the discussion of both playing and watching others play video games in online forums was mentioned regularly, perhaps providing some insight on an interesting male activity. There are several studies on video game use in children, but the literature on adult video game playing as well as watching others play, is quite limited (Weaver 3rd et al., 2009). Certainly, sedentary behavior is an independent predictor of chronic disease (Owen et al., 2010) and should be targeted along with activity to improve health. The suggestion that men not only play video games but watch others play could have important implications for intervention research.

The crowdsourcing survey brought forth many questions about physical activity. The correlation between physical activity and BMI is nothing new or unexpected, but it is interesting that many of the questions posed are related to the enjoyment of physical activity instead of strictly the practice (i.e., type, intensity, duration). Research indicates that increasing exercise improves body image in men (Davis and Cowles, 1991). Because body image came up a bit more frequently than expected, this would appear to be more of a “hook” into treatment for men than previously thought. Increasing enjoyment of physical activity may not only help to sustain the behavior in men, but may have an important feedback to body image enhancement.

Many other well-documented weight-related factors were not significantly associated with BMI in this study. Sleep has been negatively correlated with BMI in a variety of studies, but was not found to have any correlation in this investigation (Ford et al., 2014; Vgontzas et al., 2014; Zhang et al., 2015). Regular self-monitoring in the form of frequent weigh-ins or food journaling (Burke et al., 2005; Warziski et al., 2008) has also been shown to have an association with lower BMI, which was not evident in our study. Intimate personal relationships and sexual behaviors did not correlate with BMI, although previous crowdsourcing research related to BMI would suggest a strong correlation (Bongard et al., 2013). More specifically, questions about marital status and having a partner with similar diet goals were not significantly correlated with male BMI.

### 4.1 Limitations and future research

While crowdsourcing is a novel approach, there are limitations to utilizing this methodology that could be addressed in future studies. It is important to consider that not all participants answered each question. Over 300 users answered the first six questions, but the last few questions only collected 2–3 answers. While we did offer a lottery-selected financial incentive to answer all questions posted on the site at the time of each participant's initial visit, a different incentive structure to motivate participants to return to the site as more questions are added could be beneficial for further data collection. It may also be beneficial to log how long participants spent on the site, as a measure of seriousness of the survey participants. Additionally, we did not collect demographic information from crowdsourcing participants. This makes it difficult to interpret the influence of some demographic factors, such as socioeconomic status, race and age on survey responses.

We are particularly intrigued by the introduction of video games as a significant sedentary behavior with high correlation to BMI as well as the very small number of proposed questions relating to intimate personal relationships and their impact on weight. It is certainly understood in the literature that sedentary behavior is associated with higher weight, and perhaps further investigation of this population of men who participate in “gaming” is worth pursuing. Additionally, future intervention design could focus on the relationship between weight and relationship status/household roles (who does the cooking, grocery shopping, etc.) perhaps to better decipher the consistent external influences on weight management for men.

Finally, while we hoped to gather ideas related to male motivation and engagement in weight loss studies, the nature of crowdsourcing does not allow us to dictate what information we will receive from the “crowd”. Crowdsourcing allows researchers to pose questions, and while we could have perhaps guided our research question a bit differently to better target interest and enthusiasm, it was also our goal to not guide the discussion in one way or another and instead see what the “crowd” came up with. We did not end up collecting many ideas of how to better target men for weight loss interventions, but we were able to collect valuable information surrounding why men are perhaps overweight in the first place. In order to appropriately design a successful weight loss program for men, it is important to better understand the background and the behaviors to change; such as less time spent playing/watching video games or more time spent participating in organized physical activity such as team sports or planned outdoor activities.
